# Identification of a potential neutralizing linear epitope of hemagglutinin-neuraminidase in Newcastle disease virus

**DOI:** 10.1186/s12985-020-01483-y

**Published:** 2021-01-06

**Authors:** Zhongyuan Jin, Qiaolin Wei, Youkun Bi, Yongshan Li, Na Huo, Sujing Mou, Wenbin Wang, Haijin Liu, Zengqi Yang, Hongjun Chen, Sa Xiao

**Affiliations:** 1 College of Veterinary Medicine, Northwest A&F University, Yangling, 712100 Shanxi People’s Republic of China; 2 Shanghai Veterinary Research Institute, Chinese Academy of Agricultural Sciences, Shanghai, 200241 People’s Republic of China

**Keywords:** Newcastle disease virus, LaSota strain, Hemagglutinin-neuraminidase, Immunodominant epitope, Pepscan

## Abstract

**Background:**

The hemagglutinin-neuraminidase (HN) protein of Newcastle disease virus (NDV) is a major antigen that can induce protective antibodies in poultry. However, its antigenic epitopes have not been fully elucidated. Therefore, defining the linear epitopes of HN, especially neutralizing epitopes, will be useful for revealing its antigenic characterization.

**Methods:**

In this study, we analyzed B-cell immunodominant epitopes (IDEs) of the HN protein from the vaccine strain LaSota using pepscan technology with LaSota-specific chicken hyperimmune antisera. We constructed IDEs-RFP plasmids and prepared anti-IDEs peptide mouse sera to identify IDEs through immunological tests. At last, the different diluted anti-IDE antisera were used in BHK-21 cells to perform the neutralization test.

**Results:**

Five IDEs of the HN were screened and further verified by indirect immunofluorescence assays, dot blots and Western blots with NDV- and IDEs-specific antisera. All five IDEs showed good immunogenicity. IDE5 (328–342 aa) could recognize only class II NDV but did not react with the class I strain. Most of the IDEs are highly conserved among the different strains. A neutralization test in vitro showed that the peptide-specific mouse antisera of IDE4 (242–256 aa) and HN341-355, a reported neutralizing linear epitope, could partially neutralize avirulent LaSota as well as virulent strains at similar levels, suggesting that IDE4 might be a potential neutralizing linear epitope.

**Conclusion:**

The HN protein is a major protective antigen of NDV that can induce neutralizing antibodies in animals. We identified five IDEs of the HN using a pepscan approach with NDV-specific chicken hyperimmune antisera. The five IDEs could elicit specific antibodies in mice. IDE4 (242–256 aa) was identified as a novel potential neutralizing linear epitope. These results will help elucidate the antigenic epitopes of the HN and facilitate the development of NDV vaccines.

## Background

Newcastle disease (ND) is a highly contagious disease of birds that causes major economic losses in the poultry industry worldwide [1, 2]. It is caused by Newcastle disease virus (NDV), also known as avian paramyxovirus serotype 1 (APMV-1) or orthoavulavirus-1, which is currently classified into the genus *Orthoavulavirus* of subfamily *Avulavirinae* in the family *Paramyxoviridae* of order Mononegaviriales (ICTV) [3]. As an enveloped virus with a negative-sense, single-stranded RNA genome, NDV encodes the six structural proteins: nucleoprotein (NP), phosphoprotein (P), matrix protein (M), fusion protein (F), hemagglutinin-neuraminidase (HN) and large polymerase protein (L) [4]. Additionally, two other proteins, V and W, are produced by RNA editing during transcription of the viral P gene [5, 6].

HN, a surface glycoprotein of NDV, plays multiple roles in viral tropism and virulence [7]. This protein consists of a cytoplasmic domain, transmembrane region, stalk region and globular head [8] and is responsible for binding to sialic acid-containing cell surface receptors and possesses neuraminidase (NA) activity to prevent viral self-aggregation. HN also promotes the fusion activity of the F protein responsible for virus-cell and cell–cell fusion [9–11]. Importantly, HN, as a major protective antigen, can induce virus-neutralizing antibodies during NDV infection [12, 13]. Earlier studies with anti-HN monoclonal antibodies (mAbs) identified seven consecutive overlapping antigenic sites on the surface of HN [14, 15]. Among them, only site 14 (345–353 aa), referred to as HN345-353, was a linear epitope. Its corresponding mAb 14 could neutralize NDV [16]. This epitope in the infectious bursal disease virus-vectored vaccine could also induce protective antibodies in chickens against NDV [17]. In addition, a P2 (53–192 aa) region of four distinct regions of HN was identified as a dominant linear antigenic domain using a yeast surface display system that could protect most chickens from NDV challenge [18]. However, the linear antigenic epitopes of HN have not been fully elucidated. Therefore, defining the linear epitopes of HN, especially neutralizing epitopes, will be useful for revealing its antigenic characterization.

Among the methods for identifying epitopes, peptide scanning technology, such as pepscan, has been widely used due to its high sensitivity and rapidity [19]. This method utilizes a peptide synthesis technique to synthesize successive and overlapping short peptides according to the amino acid sequence of the target protein molecule and then reacts these synthetic peptides one by one with the antibody to determine the position of the peptide segment of the antibody recognition epitope [20]. Recently, this technology was also successfully used for a comprehensive antigenic analysis of viral proteins [21, 22]. In NDV, two immunodominant B-cell linear epitopes of the viral M protein were identified by using this pepscan approach [23].

In conclusion, we defined five linear immunodominant epitopes (IDE) of the HN protein in the vaccine strain LaSota using pepscan and further analyzed their antigenicity and immunogenicity. A neutralization test indicated that IDE4 (242–256aa) could induce a neutralizing antibody against NDV. This molecule might be a potential neutralizing epitope of HN. Our study can help elucidate the antigenic epitopes of HN and the development of an epitope vaccine for NDV.

## Materials and methods

### Cells and viruses

The chicken embryonic fibroblast cell line DF-1, BHK-21 and HEK293T were purchased from the ATCC (USA) and were grown in Dulbecco’s modified Eagle’s medium (DMEM) (Gibco, Thermo, Grand Island, NY, USA) supplemented with 10% (v/v) fetal bovine serum (FBS) (Gibco, USA) and 1% penicillin–streptomycin (100x, Macgene, China) in a humidified 5% CO_2_ atmosphere at 37 °C.

The F48E9 and LaSota strains of NDV were obtained from the China Institute of Veterinary Drug Control. The class II strains SX10 and JS17 and the class I strain QH-1 were isolated from China by our laboratory. All viruses were propagated in 9- to 11-day-old specific-pathogen-free chicken embryonated eggs. Fresh allantoic fluid was harvested from embryonated eggs dead between 24 and 120 h after inoculation and kept at − 80 °C.

### Pepscan analysis

The pepscan analysis was performed by the Novasnow Science & Technology China with anti-LaSota hyperimmune chicken serum as described previously [23]. The HN protein sequences were linked and elongated with neutral GSGSGSG linkers at the C- and N-termini to avoid truncated peptides. The elongated antigen sequences were translated into 15 amino acid peptides with a peptide–peptide overlap of 14 amino acids. The HA (YPYDVPDYAG,100 spots) and c-myc (EQKLISEEDL, 100 spots) were used as control peptides.After 15 min of preswelling in washing buffer and 30 min of incubation in blocking buffer, peptide microarrays were initially incubated with the secondary antibody goat antichicken IgG (H + L) DyLight680 (1:5000) and with control antibody mouse monoclonal anti-HA (12CA5) DyLight800 (1:2000) for 45 min at 37 °C to analyze background interactions with the antigen-derived peptides. Then, the peptide microarray analysis was performed with anti-LaSota chicken hyperimmune serum diluted at 1:500 in incubation buffer for 16 h at 4 °C and shaking at 140 rpm. The LI-COR Odyssey Imaging System was used to scan the preprocessed peptide microarrays at scanning intensities of 7/7 (red = 700 nm/green = 800 nm).

### Preparation of anti-LaSota chicken hyperimmune sera, anti-LaSota mouse hyperimmune sera and anti-IDE peptide mouse sera

Four-week-old specific pathogen-free (SPF) white leghorn chickens (SAIS Poultry) were inoculated through the intraocular–nasal route with 0.2 ml per chicken of LaSota live vaccine on day one. Then, the chickens were immunized with 0.2 ml per chicken of inactivated vaccine through subcutaneous injection on days 14 and 28. Blood samples were collected on day 35 [23].

Six-week-old SPF female BALB/c mice (purchased from DaShuo, China) were subcutaneously immunized with 0.2 ml per mouse with inactivated LaSota vaccine (Yebio Qingdao, China) at days 1, 14 and 28. The sera were collected at day 35 and kept at − 80 °C.

For preparation of an anti-HN guinea pig polyclonal antibody (pAb), one-month-old guinea pigs were immunized with HN, which was obtained by prokaryotic expression, at 50 μg per animal. The guinea pigs were subcutaneously injected with a mixture of the protein and complete Freund’s adjuvant (Sigma). The animals were subcutaneously injected with a mixture of the protein and incomplete Freund’s adjuvant (Sigma) at 14 and 28 days. After the guinea pigs were anesthetized with ketamine and xylazine, their antisera were collected and stored at − 80 °C [24].

Due to 15 amino acid peptides are usually too short to provoke a sufficient immune response, the IDE peptides and an HN341-355 peptide of the previously reported HN neutralizing epitope 346–353 [16], were synthesized and conjugated with KLH by Ontores Biotechnology (Shanghai, China). Each peptide was dissolved to 1 mg/ml with phosphate-buffered saline (PBS). Four six-week-old SPF female BALB/c mice with peptide emulsified by complete Freund’s adjuvant (Sigma) were subcutaneously immunized with 50 μg per mouse. The mice were boosted twice with the peptide emulsified by incomplete Freund’s adjuvant (Sigma) at day 14 and 28. Finally, blood samples were collected at day 38. The antisera of five IDEs and HN341-355 were prepared.

### Construction of the IDE-RFP plasmids

Five IDEs and HN341-355 fusion with red fluorescent protein (RFP) were constructed by overlapping PCR. Upstream primers were combined with IDE sequences and the 3′ terminus of the RFP gene. The downstream primer was located at the 5′ terminus of the RFP gene. The primers are listed in Additional file 1: Table S1. After overlapping PCR, the IDE-RFP and HN341-355-RFP fragments were digested by the restriction enzymes *Xho*I and *Bgl*II and ligated into the pCAGGS vector. The products were confirmed by DNA sequencing.

### Western blot analysis

HEK293T cells were cultured at approximately 70–80% confluence in 12-well plates and transfected with 2 μg each of expression plasmids (IDEs-RFP and HN341-355-RFP) by using Tuborfect Transfection Reagent (Thermo Fisher Scientific, USA) in accordance with the manufacturer’s guidelines. The cells were harvested at 48 h post transfection. The cell samples were washed with PBS and lysed with lysis buffer (2% SDS, 10% glycerin, 5% β-mercaptoethanol and 0.1% bromophenol blue). The lysates were collected in 1.5 ml tubes on ice for 30 min and centrifuged at 12,000 rpm at 4 °C for 15 min for clarification. After incubation at 100 °C for 10 min, an equal amount of these prepared samples was subjected to sodium dodecyl sulfate–polyacrylamide gel electrophoresis (SDS-PAGE), transferred onto a nitrocellulose blotting membrane (Millipore, USA) and blocked in 10% skim milk in PBS at 4 °C overnight. After the membrane was washed with PBST (PBS containing 0.5% Tween-20), it was incubated with primary antibodies for 2 h at 37 °C. After washing, the protein bands were detected with an HRP-conjugated IgG antibody (Abcam). Finally, after three more washes, the antibody-antigen complex was exposed with a chemiluminescence (ECL) reagent solution kit (Share-bio Biotechnology, China) by using a Tanon 5200 multichemiluminescence image analysis system (China).

### Dot blot

Each synthesized peptide (1 µg) was dropped onto the nitrocellulose membrane and incubated at 37 °C for 30 min. The membrane was blocked with 5% skim milk at 37 °C for 2 h. After the membrane was washed three times with TBS, it was incubated with the 1:2000 anti-IDE mouse antibody diluted with TBS at 37 °C for 1 h. After the membrane was washed with TBST, it was incubated with the corresponding HRP-conjugated goat anti-mouse secondary antibody. The ECL peroxidase substrate (Millipore) was used for detection.

### Indirect immunofluorescence assay (IFA)

BHK-21 cells were cultured at approximately 60–70% confluence in 48-well plates and transfected with 1 μg each of the expression plasmids by using Tuborfect Transfection Reagent (Thermo Fisher Scientific, USA) in accordance with the manufacturer’s guidelines. DF-1 cells were cultured at 60% confluence in 48-cell plates and infected with LaSota at an MOI of 1. The cells were washed with PBS and fixed with 4% methanol for 10 min at room temperature. After the cells were washed three times with PBS, they were blocked with 1% BSA in PBS at 37 °C for 2 h, and then, the cells were inoculated with a primary antibody at 4 °C overnight. After the cells were washed thrice with PBST, they were incubated with secondary antibody conjugated FITC (Abcam) in a moist container in the dark at 37 °C. After the cells were washed thrice with PBST, they were stained with DAPI (0.1 μg/ml) for 8 min at room temperature. Finally, fluorescent images were visualized and captured on a confocal fluorescence microscope (Olympus, Japan).

### Enzyme-linked immunosorbent assay

One hundred nanograms of synthetic peptides was coated on ELISA plates in 100 μl of 0.05 M carbonated bicarbonate (pH 9.6) by overnight incubation at 4 °C. After the samples were washed four times with PBST, the plate was blocked with 10% skim milk at 37 °C for 2 h and washed with PBST. A total of 100 μl of antisera of the IDEs diluted 1:200, 1:400, 1:800, 1:1600, 1:3200 and 1:6400 with PBS was added to the indicated wells and incubated at 37 °C for 2 h. The anti-LaSota mouse serum was diluted 1:500 with PBS. The plate was washed again. Horseradish peroxidase (HRP)-conjugated goat anti-mouse IgG (Abcam) was added to each well and incubated at 37 °C for 45 min. After another wash, 100 μl of TMB substrate was added to each well. The reaction was stopped with 50 μl of 0.5 M H_2_SO_4_ after incubation at room temperature for 30 min. The OD_450_ value of each well was immediately read with a Microplate Absorbance Reader (Bio-Rad, USA).

### Neutralization assay

The different diluted antisera (anti-IDE, anti-HN341-355 mouse sera, HN guinea pig polyclonal antiserum) and normal BALB/C mouse serum as controls were used in BHK-21 cells to perform the neutralization test. Twelve microliters of the antisera treated at 56 °C for 30 min was serially diluted twofold from 1/4 to 1/512. Then, the diluted antisera were mixed with three viruses, LaSota (50 μl, 200 TCID_50_), JS17 (50 μl, 50 TCID_50_) and F48E9 (50 μl, 10 TCID_50_), and incubated at 37 °C for 2 h in a 96-well plate. Subsequently, 100 μl of the mixtures was transferred to BHK-21 cells in a 96-well plate and incubated at 37 °C for 1 h. After three washes with PBS, DMEM containing 2% FBS was added. After incubation for 12 h, the LaSota- and JS17-infected BHK-21 plates were fixed for 20 min with methanol. After the plates were washed again, the HN pAb from guinea pigs and the anti-NDV HN monoclonal antibody were used to identify the number of infected cells by IFA. The number of infected cells was calculated and analyzed by ImageJ. Data are presented as the average of triplicates. The F48E9-infected BHK-21 cells were calculated by the average number of syncytia.

### Statistical analysis

Statistical analyses were performed using GraphPad Prism 5. Data from three independent experiments are presented as the mean ± standard deviation (SD) from triplicate samples (*n* = 3); *P* < 0.05 with Student’s t-test was considered to be statistically significant. Statistical significance was set at *P* < 0.05 (*), *P* < 0.01 (**), and *P* < 0.001 (***).

## Results

### Scanning B-cell linear epitopes of the HN

**Fig. 1:**
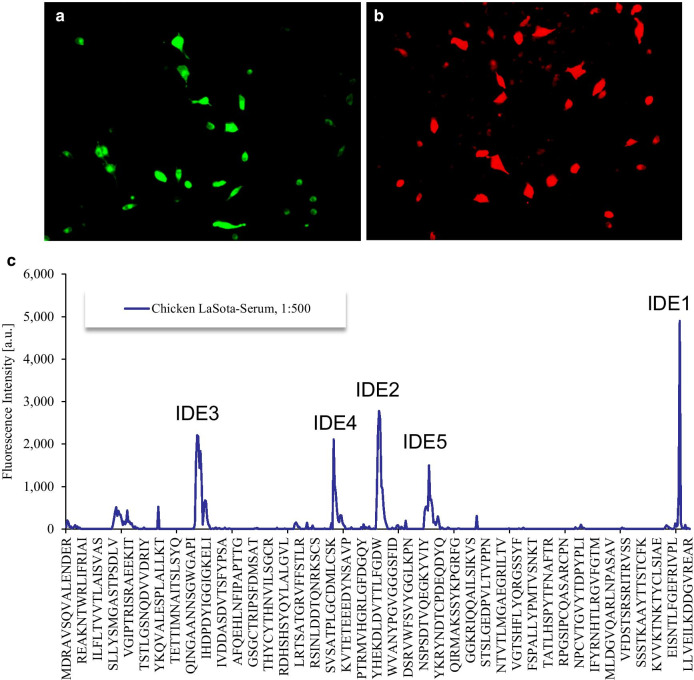
Pepscan analysis of HN with NDV-specific antisera. BHK-21 cells were transfected with the pCAGGS-HN plasmid. After transfection for 48 h, the cells were fixed and incubated with NDV-specific chicken hyperimmune antisera (**a**) and mouse antisera (**b**). Subsequently, the cells were incubated with an FITC-conjugated rabbit anti-chicken antibody and a goat anti-mouse antibody. The cells were observed by fluorescence microscopy. Scale bar = 50 µm. **c** Signal diagram of the pepscan. The data quantification was followed by generation of intensity plots. The x-axis indicates the peptide sequences of the HN protein from the N-terminus (left) to the C-terminus (right). The five highly reactive peaks are shown as IDE1-5

HN as a glycosylated transmembrane protein of NDV can induce a protective humoral response against viruses in avian species. First, we examined whether the HN protein can be recognized by NDV-specific chicken antisera. BHK-21 cells were transfected with the HN protein, which was analyzed by immunofluorescence assays using hyperimmune NDV-specific antisera prepared by LaSota vaccination. The antisera could recognize the expressed HN protein (Fig. 1a, b). Then, NDV-specific chicken antisera were used to scan the B-cell linear epitopes of the HN protein by the pepscan approach. The data showed five epitopes with high serum response intensity, which were selected as immunodominant epitope (IDE) candidates (Fig. 1c). Each IDE contained fifteen amino acids (Table 1).

**Table 1:** The HN epitope candidates by pepscan

Epitope	Sequence (15 aa)	fluorescence intensity	Deviation (%)
IDE1	554-GEFRIVPLLVEILKD-568	4897.8	27.5
IDE2	283-EKDLDVTTLFGDWVA-297	2778.3	3.7
IDE3	119-NNSGWGAPIHDPDYI-133	2209	1.4
IDE4	242-ATPLGCDMLCSKVTE-256	2110.5	15.7
IDE5	328-TVQEGKYVIYKRYND-342	1497.5	5.9

*aa* Amino acid

### Verification of the immunodominant epitopes

**Fig. 2:**
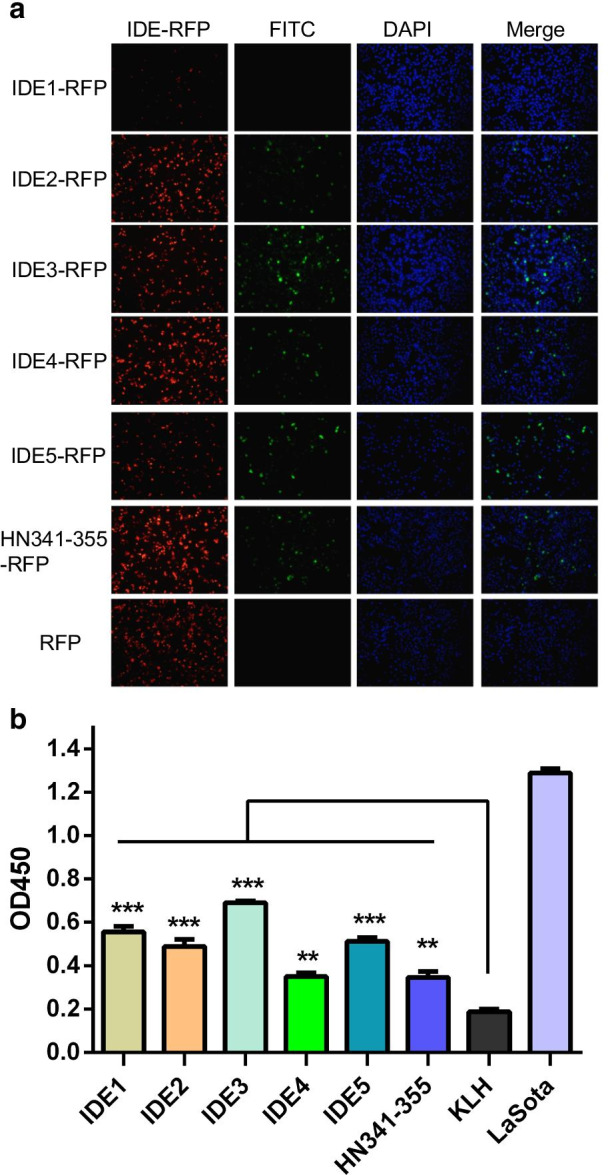
Identification of IDEs. **a** IFA analysis of IDEs. BHK-21 cells were transfected with IDE-RFP constructs. After 48 h, the cells were fixed and incubated with HN-specific guinea pig pAb and subsequently incubated with an FITC-conjugated goat anti-mouse antibody. The cell nuclei were stained with 4′,6′-diamidino-2-phenylindole (DAPI). The cells were observed by fluorescence microscopy. Scale bar = 50 µm. RFP (red), IDEs and HN341-355 (green) as well as the cell nucleus (blue) are shown. **b** ELISA data of the IDEs. The 96-well plates were coated with 200 ng of each synthetic IDE and HN341-355 peptide and incubated with NDV-specific mouse antisera. The purified LaSota virus was used as a positive control. KLH was used as a negative control. **P < 0.01; ***P < 0.001

For verification of the IDEs, the five epitope candidates and HN341-355 were fused to RFP in a eukaryotic expression vector. All RFP-IDEs and RFP-HN341-355 were recognized by anti-HN pAb from guinea pigs in the transfected BHK-21 cells, whereas RFP-IDE1 showed lower reactivity with the antisera (Fig. 2a). Furthermore, the synthetic peptides of the five IDEs and HN341-355 conjugated with KLH were tested by ELISAs. All peptides presented positive reactions comparing with negtive control KLH (Fig. 2b). The IDEs could not be detected with NDV-specific antisera by dot blots. The results indicated that the five IDEs and HN341-355 could be recognized by the HN-specific pAb.

### Immunogenicity of IDEs

**Fig. 3:**
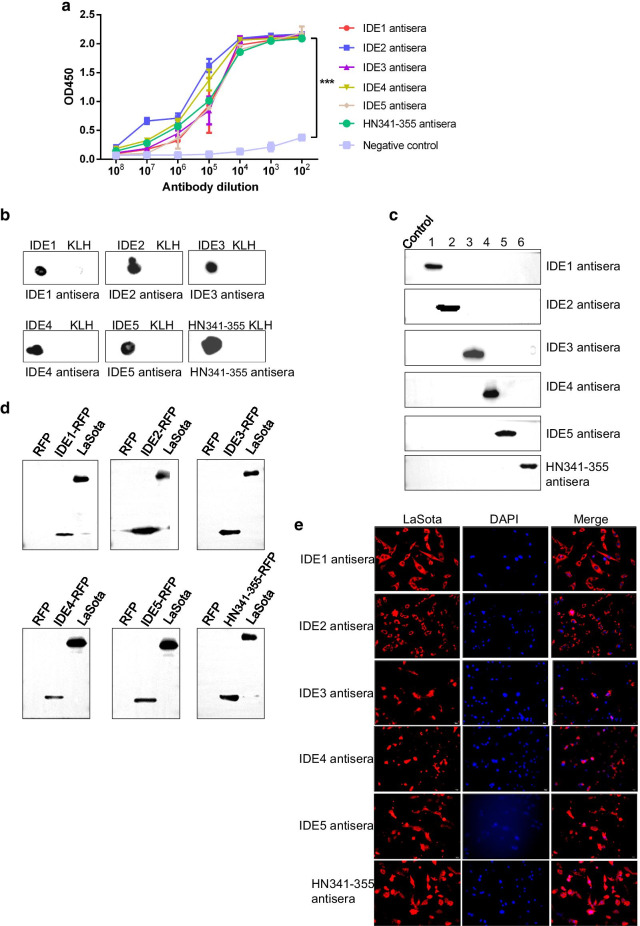
Immunogenicity of the IDE peptides. **a** The antibody titers of anti-IDE and anti-HN341-355 mouse sera by ELISAs. The nonimmunized mouse serum was used as a negative control. Representative data, shown as the means ± SDs (*n* = 3), were analyzed by two-tailed Student’s *t* test. ***P < 0.001. **b** Dot blot analysis. The IDE synthetic peptides (2 μg) were dropped on a nitrocellulose membrane and incubated with the IDE mouse antisera. KLH was used as a negative control. **c** The cross-reactivity of IDE mouse antisera by Western blot. RFP was used as a negative control. **d** The detection of RFP-IDEs by Western blot. The purified LaSota virus was used as a positive control. Single RFP expression was used as a negative control. **e** IDE mouse antisera recognizing LaSota virus. DF-1 cells were infected with LaSota at an MOI of 1. The cells were fixed at 24 h post-infection and analyzed by IFA with IDE mouse antisera

To analyze the immunogenicity of the IDEs and HN341-355, we immunized the mice with the synthetic peptides. The antisera were prepared for serological analysis. The five IDEs and HN341-355 induced high antibody titers, as shown by ELISAs (Fig. 3a), and recognized the peptides by dot blots (Fig. 3b). Each antiserum did not cross-react with other IDEs (Fig. 3c), indicating the specific recognition of these antisera. Furthermore, these antisera strongly reacted with IDE-RFP and HN341-355-RFP by Western blots (Fig. 3d). These results indicated that the IDEs and HN341-355 could specifically elicit a humoral response in animals. In addition, these antisera could recognize HN in the LaSota-infected cells (Fig. 3e). IDE1 and IDE2 clearly appeared in the cytoplasm, while other IDEs likely existed in the whole cells, suggesting that they might be related to the position of the IDEs in the HN structure. These results indicated that these five IDE peptides as well as the HN341-355 peptide could specifically elicit humoral responses.

### Identification of the neutralizing epitope

**Fig. 4:**
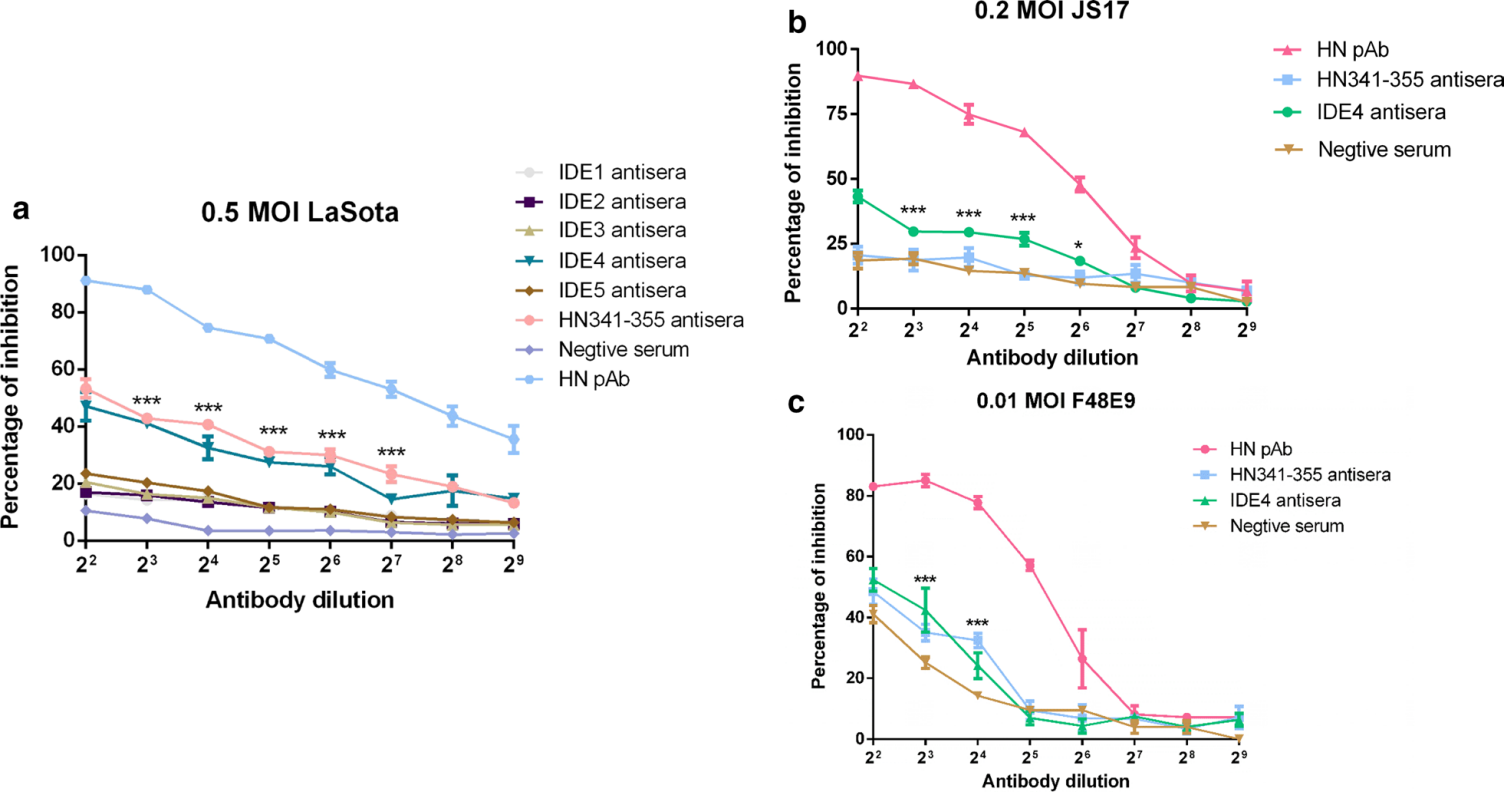
Virus neutralization of the IDE-specific antisera. The IDE mouse antisera were serially diluted in 96-well plates and incubated with LaSota at 200 TCID_50_ (**a**), JS17 at 50 TCID_50_ (**b**) and F48E9 at 10 TCID_50_ (**c**). **a** Virus-neutralizing activities against LaSota. Twelve hours after infection, the cells were detected by IFA with the anti-HN guinea pig pAb. The number of positive cells and the inhibition rate were calculated. Percentage of inhibition was determined as follows: 100%—percent of infected cells/percent of the infected cells in negative serum for each dilution. **b** Virus-neutralizing activities against JS17. The cells were detected by IFA with anti-NDV HN monoclonal antibody. The calculation method is the same as above. **c** Virus-neutralizing activities against F48E9. The number of syncytia was observed at different times after virus infection. Percentage of inhibition was determined as follows: 100%—the average number of syncytia/the average number of syncytia in negative serum for each dilution. Representative data of the IDEs and HN341–355 antisera compared to the negative serum, shown as the means ± SDs (*n* = 3), were analyzed by two-tailed Student’s *t* test. *P < 0.05; ***P < 0.001

HN antibodies possessing neutralizing epitopes can effectively block NDV infection. Residues 346–353 aa of HN were defined as a neutralizing epitope [16]. To investigate whether the IDE-specific antisera had virus-neutralizing activity, we performed a virus neutralization assay in BHK-21 cells. The anti-HN guinea pig pAb could significantly inhibit LaSota infection at high levels compared to the negative serum. The viral inhibition still reached 53%, even though the dilution of the pAb was 2^7^. Both antisera of IDE4 and HN341-355 showed partial inhibition of LaSota infection at similar levels compared to the negative serum. The neutralizing levels of the IDE4 antisera gradually decreased with inhibition rates of 47%, 41%, 31%, 27%, 26%, and 14% at dilutions of 2^2^ to 2^7^. Other antisera of the IDEs and negative serum could not prevent virus infection (Fig. 4a). These data indicated that the antisera of IDE4 and HN341-355 showed partial virus-neutralizing activities against avirulent LaSota. Moreover, we tested whether the IDE4 antisera neutralized the highly virulent strains JS/17 and F48E9 in BHK-21 cells. The IDE4 antisera showed slight inhibition of the JS17 virus at dilutions of 2^2^ to 2^4^ compared to the negative serum, but the HN341-355 antisera did not inhibit the virus as did the negative serum (Fig. 4b). The IDE4 antisera slightly inhibited the F48E9 virus compared to the negative serum (Fig. 4c). Other antisera of the IDEs could not prevent F48E9 infection. Taken together, these results suggested that IDE4 might be a potential virus-neutralizing epitope of viral HN for NDV.

### Conservation of the IDEs in different strains

**Fig. 5:**
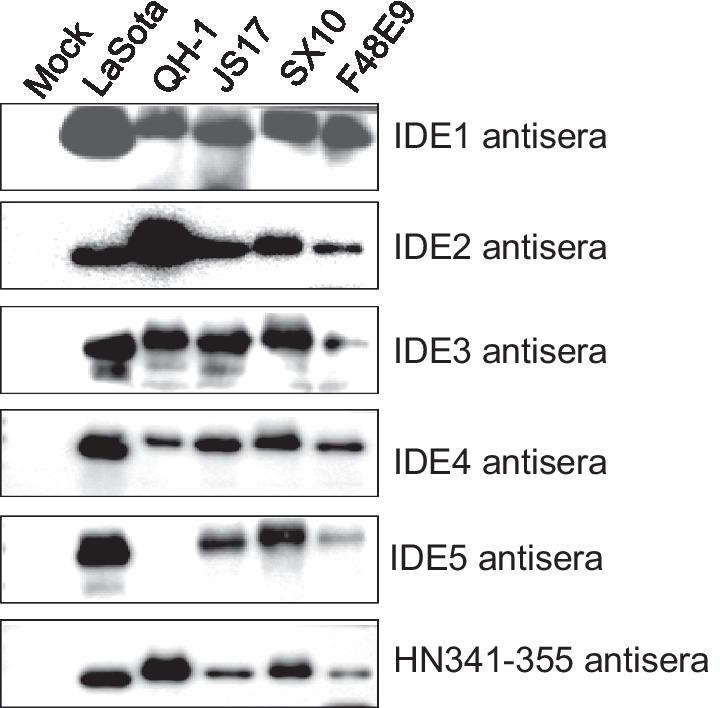
Serological recognition of the HN protein in multiple NDV strains by IDE mouse antisera. The five purified NDV strains were diluted for Western blot analysis with IDE antisera and HN341–355 antisera. Normal allantoic fluid was used as a mock infection control

To examine whether the five IDEs were conserved in different strains of NDV, we analyzed the five strains by Western blots. The antisera of four IDEs as well as HN341-355 in addition to IDE5 could recognize all five viruses. The IDE5 antisera could recognize LaSota, JS17, SX10 and F48E9 of the class II strains but did not react with QH-1 of the class I strain (Fig. 5), suggesting that IDE5 could be used to serologically differentiate class I and class II NDV strains. Moreover, the multiple amino acid sequence alignment of the HN in these five strains showed that IDE1 and IDE4 had the highest amino acid identities (Additional file 1: Table 2). IDE5 had the lowest amino acid identity in the QH-1 strain, which was consistent with the Western blot results. The amino acids of IDE4 in genotypes I to XIII of class II were also shown to be highly conserved (data not shown). These results indicated that IDE4 was a highly conserved epitope for class II strains.

### Three-dimensional localization of the IDEs in the HN protein structure

**Fig. 6:**
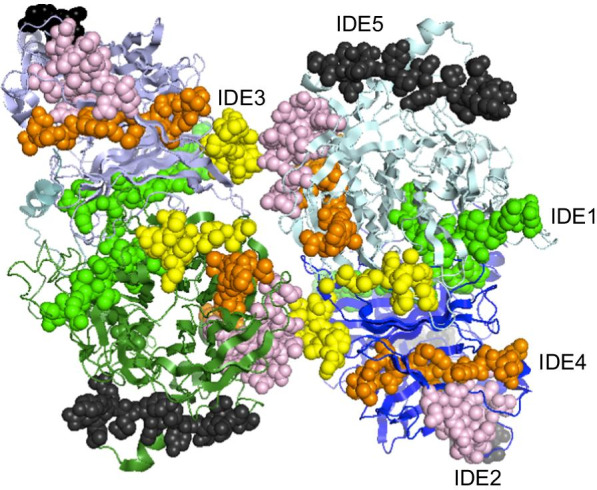
Three-dimensional localization of the IDE1-IDE5 in the HN protein structure. The spatial structure of HN protein was visualized using PyMol (Schrodinger, Inc.). The HN presented as a homotetramer, and the five IDEs were indicated in the structure to show their location in different colors. Green represents IDE1 (554–568 aa). Pink represents IDE2 (283–297 aa). Yellow represents IDE3 (119–133 aa). Orange represents IDE4 (242–256 aa). Black represents IDE5 (328–342 aa)

In order to show the position of IDEs in HN more directly, we established the crystal model of LaSota HN protein using SWISS-MODEL and performed a three-dimensional structure analysis, as shown in Fig. 6. IDE1, IDE2, IDE4 and IDE5 were located at the surface of HN protein, which suggest that they could feasibly form linear antigenic epitopes and induce to produce protective antibodies or neutralizing antibodies. However, IDE3 was located in the transmembrane domain, we speculate that it may be related to the membrane fusion activity induced by HN protein, but has little effect on the production of neutralizing antibodies.

## Discussion

The HN protein is a major protective antigen of NDV that can induce neutralizing antibodies in animals [25, 26]. Its B-cell antigenic epitopes have not been fully elucidated. To investigate the epitopes of this protein, we analyzed its linear epitopes from the conventional vaccine strain LaSota using pepscan technology. The five distinct IDEs of HN were verified and showed good immunogenicity in animals. IDE3 is located in the transmembrane domain, and the other IDEs are distributed in the global head of the HN structure. Among them, IDE4 (242–256aa) was identified as a novel neutralizing linear epitope.

HN341-355 is the only known linear neutralizing epitope of HN [16]. In our study, IDE4 was found to be another linear neutralizing epitope. However, the antisera of IDE4 and HN341-355 did not show a high level of virus-neutralizing activity. In fact, most HN antibodies with neutralizing activity recognize conformational epitopes [15, 25]. The complete neutralization of NDV requires the synergy of mAbs at different neutralization sites in the HN protein [27]. Furthermore, the viral F protein is necessary for NDV infection of host cells [28]. The neutralizing epitopes of F can induce antibodies to block viral infection [29]. The protection provided by the simultaneous presence of anti-HN and anti-F serum was significantly greater than that afforded by either treatment alone or comparable to that of anti-whole NDV serum [30]. HN341-355 only provides partial protection against NDV challenge in immunized chickens [17].

The IDE4 antisera, similar to the HN341-355 antisera, showed partial inhibition of LaSota and slight inhibition of virulent viruses in the virus neutralization assay. The neutralizing activity of the IDE4 antisera to avirulent virus seems higher than that of the virulent strains JS17 and F48E9, even though the amino acid sequence of IDE4 was highly conserved among the three strains. This result might be due to the difference in avirulent and virulent envelope proteins. The amino acid identities of HN in the avirulent LaSota compared to the virulent viruses JS17 and F48E9 are quite different at 88% and 91%, respectively. In addition to HN, the viral F protein, another protective antigen of NDV, is responsible for inducing virus-neutralizing antibodies [29]. The amino acid identities of F for LaSota to JS17 and F48E9 are 88% and 92%, respectively. The differences in the HN and F proteins between avirulent and virulent strains may cause the different infectivity and virulence of the lentogenic and velogenic viruses. Therefore, the ability of a single antibody against one neutralizing epitope of HN to block virus infection may be different between avirulent and virulent viruses. A complete understanding of the relationship between antibody recognition and virulence requires a complete mapping of each of the amino acids of the HN contributing to the epitope as previously described [31].

Pepscan is a widely used method for identifying epitopes [20], but it still has some deficiencies, for example, the short linear epitope is often not recognized with high affinity by antibodies using pepscan, resulting in low accuracy for scanned data [32]. In our study, the peptides of six epitopes conjugated to KLH were synthesized. They were detected by anti-IDE mouse antisera in dot blot analysis but not detected with NDV-specific chicken antisera (data not shown), which were used for pepscan. However, the synthetic peptides coated on ELISA plates could bind with NDV-specific chicken antisera in ELISAs. One possibility is that the IDE peptides blotted on the membrane may not be fully recognized by the NDV-specific chicken antisera in the dot blots. The sensitivity and specificity of the mouse antisera were higher than those of chicken antisera in ELISAs. In addition, the RFP-IDE and HN341-355 constructs in the transfected cells could not react with the NDV-specific chicken antisera, but they were recognized by anti-HN guinea pig pAb. The nonrecognition of NDV-specific chicken antisera by IDE peptides is unclear. This finding is likely related to the immune process of host species, which result in different affinities of the antibodies.

## Conclusion

In this study, we identified five IDEs of the HN in NDV using a pepscan approach. The immunized mice with five IDE peptides produced the IDE-specific antibodies which did not cross-react each other. The IDE4 and HN341-355, a previously reported neutralizing linear epitope, present similar levels of virus-neutralizing activity by in vitro assay. The amino acid sequence of IDE4 (242–256aa) as a novel neutralizing epitope has high conservation in the HN of NDV strains. These results will help to understand the antigenic characteristics of HN and develop the subunit vaccines of NDV.

## Supplementary Information

**Additional file 1.** **Table S1.** The primers for IDE-RFP construction. **Table S2.** Alignment of live IDEs and HN341-355.

## Data Availability

All data generated or analysed during this study are included in this published article and its supplementary information files.

## Abbreviations

NDV: Newcastle disease virus
HN: Hemagglutinin-neuraminidase
IDE: Immunodominant epitopes
SPF: Specific pathogen-free
pAb: Polyclonal antibody
