# Supplementary material for: Identification of a potential neutralizing linear epitope of hemagglutinin-neuraminidase in Newcastle disease virus
Source: Virol J. 2021 Jan 6;18:8. doi: 10.1186/s12985-020-01483-y (PMC7789432; doi:10.1186/s12985-020-01483-y)
Supplement: Supplementary file 1 — Additional file 1. Table S1. The primers for IDE-RFP construction. Table S2. Alignment of live IDEs and HN341-355. [file 12985_2020_1483_MOESM1_ESM.doc]

**Supplementary Table 1. The primers for IDE-RFP construction.**

| Name | Sequence(5’-3’) |
| --- | --- |
| IDE1-F | CCGCTCGAGatgggagaattcagaatcgtcccgttactagttgagatcctcaaagatatgagcgagctgatcaaggag |
| IDE2-F | CCGCTCGAGatggaaaaggacctagatgtcacaacattattcggggactgggtggccatgagcgagctgatcaaggag |
| IDE3-F | CCGCTCGAGatgaacaacagtgggtggggggcacctatccatgacccagattatataatgagcgagctgatcaaggag |
| IDE4-F | CCGCTCGAGatggcaactcccctgggttgtgatatgctgtgctcgaaagtcacggagatgagcgagctgatcaaggag |
| IDE5-F | CCGCTCGAGatgactgtacaggaagggaaatatgtgatatacaagcgatacaatgacatgagcgagctgatcaaggag |
| HN(341-355)-F | CCGCTCGAGatgaatgacacatgcccagatgagcaagactaccagattcgaatggccatgagcgagctgatcaaggag |
| IDE-R | GGAAGATCTttatcatctgtgccccagtttgctaggg |

**Supplementary Table 2. Alignment of live IDEs and HN341-355.**

|  | IDE1 554-568aa | | | | | | | | | | | | | | | |  |
| --- | --- | --- | --- | --- | --- | --- | --- | --- | --- | --- | --- | --- | --- | --- | --- | --- | --- |
| Strain | Genotype | **554** | **555** | **556** | **557** | **558** | **559** | **560** | **561** | **562** | **563** | **564** | **565** | **566** | **567** | **568** | |
| **LaSota** | **II** | **G** | **E** | **F** | **R** | **I** | **V** | **P** | **L** | **L** | **V** | **E** | **I** | **L** | **K** | **D** | |
| **QH-1** | **I** | **-** | **-** | **-** | **-** | **-** | **-** | **-** | **-** | **-** | **-** | **-** | **-** | **-** | **-** | **R** | |
| **JS/17** | **VII** | **-** | **-** | **-** | **-** | **-** | **-** | **-** | **-** | **-** | **-** | **-** | **-** | **-** | **-** | **-** | |
| **SX10** | **VI** | **-** | **-** | **-** | **-** | **-** | **-** | **-** | **-** | **-** | **-** | **-** | **-** | **-** | **-** | **-** | |
| **F48E9** | **IX** | **-** | **-** | **-** | **-** | **-** | **-** | **-** | **-** | **-** | **-** | **-** | **-** | **-** | **-** | **-** | |

|  | IDE2 283-297aa | | | | | | | | | | | | | | | |
| --- | --- | --- | --- | --- | --- | --- | --- | --- | --- | --- | --- | --- | --- | --- | --- | --- |
| Strain | Genotype | **283** | **284** | **285** | **286** | **287** | **288** | **289** | **290** | **291** | **292** | **293** | **294** | **295** | **296** | **297** |
| **LaSota** | **II** | **E** | **K** | **D** | **L** | **D** | **V** | **T** | **T** | **L** | **F** | **G** | **D** | **W** | **V** | **A** |
| **QH-1** | **I** | **-** | **R** | **-** | **-** | **-** | **-** | **R** | **-** | **-** | **-** | **-** | **-** | **-** | **-** | **-** |
| **JS/17** | **VII** | **-** | **-** | **-** | **-** | **-** | **T** | **-** | **V** | **-** | **-** | **K** | **-** | **-** | **-** | **-** |
| **SX10** | **VI** | **-** | **-** | **-** | **-** | **-** | **T** | **-** | **V** | **-** | **-** | **K** | **-** | **-** | **-** | **-** |
| **F48E9** | **IX** | **-** | **-** | **-** | **-** | **-** | **-** | **-** | **-** | **-** | **-** | **-** | **-** | **-** | **-** | **-** |

|  | IDE3 119-133aa | | | | | | | | | | | | | | | |
| --- | --- | --- | --- | --- | --- | --- | --- | --- | --- | --- | --- | --- | --- | --- | --- | --- |
| Strain | Genotype | **119** | **120** | **121** | **122** | **123** | **124** | **125** | **126** | **127** | **128** | **129** | **130** | **131** | **132** | **133** |
| **LaSota** | **II** | **N** | **N** | **S** | **G** | **W** | **G** | **A** | **P** | **I** | **H** | **D** | **P** | **D** | **Y** | **I** |
| **QH-1** | **I** | **-** | **S** | **-** | **-** | **C** | **-** | **-** | **-** | **V** | **-** | **-** | **-** | **-** | **-** | **-** |
| **JS/17** | **VII** | **-** | **-** | **-** | **-** | **C** | **-** | **-** | **-** | **V** | **-** | **-** | **-** | **-** | **-** | **-** |
| **SX10** | **VI** | **-** | **-** | **-** | **-** | **C** | **-** | **-** | **-** | **V** | **-** | **-** | **-** | **-** | **-** | **-** |
| **F48E9** | **IX** | **-** | **-** | **-** | **-** | **C** | **-** | **-** | **-** | **V** | **-** | **-** | **-** | **-** | **-** | **-** |

|  | IDE4 242-256aa | | | | | | | | | | | | | | | |
| --- | --- | --- | --- | --- | --- | --- | --- | --- | --- | --- | --- | --- | --- | --- | --- | --- |
| Strain | Genotype | **242** | **243** | **244** | **245** | **246** | **247** | **248** | **249** | **250** | **251** | **252** | **253** | **254** | **255** | **256** |
| **LaSota** | **II** | **A** | **T** | **P** | **L** | **G** | **C** | **D** | **M** | **L** | **C** | **S** | **K** | **V** | **T** | **E** |
| **QH-1** | **I** | **-** | **-** | **-** | **-** | **-** | **-** | **-** | **-** | **-** | **-** | **-** | **-** | **-** | **-** | **-** |
| **JS/17** | **VII** | **-** | **-** | **-** | **-** | **-** | **-** | **-** | **-** | **-** | **-** | **-** | **-** | **-** | **-** | **G** |
| **SX10** | **VI** | **-** | **-** | **-** | **-** | **-** | **-** | **-** | **-** | **-** | **-** | **-** | **-** | **-** | **-** | **-** |
| **F48E9** | **IX** | **-** | **-** | **-** | **-** | **-** | **-** | **-** | **-** | **-** | **-** | **-** | **-** | **-** | **-** | **-** |

|  | IDE5 328-342aa | | | | | | | | | | | | | | | |
| --- | --- | --- | --- | --- | --- | --- | --- | --- | --- | --- | --- | --- | --- | --- | --- | --- |
| Strain | Genotype | **328** | **329** | **330** | **331** | **332** | **333** | **334** | **335** | **336** | **337** | **338** | **339** | **340** | **341** | **342** |
| **LaSota** | **II** | **T** | **V** | **Q** | **E** | **G** | **K** | **Y** | **V** | **I** | **Y** | **K** | **R** | **Y** | **N** | **D** |
| **QH-1** | **I** | **K** | **R** | **-** | **-** | **-** | **Q** | **-** | **A** | **-** | **-** | **E** | **-** | **-** | **-** | **-** |
| **JS/17** | **VII** | **-** | **A** | **-** | **-** | **-** | **-** | **-** | **-** | **-** | **-** | **-** | **-** | **H** | **-** | **N** |
| **SX10** | **VI** | **-** | **T** | **-** | **-** | **-** | **-** | **-** | **-** | **-** | **-** | **-** | **-** | **-** | **-** | **N** |
| **F48E9** | **IX** | **-** | **A** | **-** | **-** | **-** | **R** | **-** | **-** | **-** | **F** | **-** | **-** | **-** | **-** | **-** |

|  | HN341-355 341-355aa | | | | | | | | | | | | | | | |
| --- | --- | --- | --- | --- | --- | --- | --- | --- | --- | --- | --- | --- | --- | --- | --- | --- |
| Strain | Genotype | **341** | **342** | **343** | **344** | **345** | **346** | **347** | **348** | **349** | **350** | **351** | **352** | **353** | **354** | **355** |
| **LaSota** | **II** | **N** | **D** | **T** | **C** | **P** | **D** | **E** | **Q** | **D** | **Y** | **Q** | **I** | **R** | **M** | **A** |
| **QH-1** | **I** | **-** | **-** | **-** | **-** | **-** | **-** | **D** | **H** | **E** | **-** | **-** | **V** | **-** | **-** | **-** |
| **JS/17** | **VII** | **-** | **N** | **-** | **-** | **-** | **-** | **-** | **-** | **-** | **-** | **-** | **-** | **-** | **-** | **-** |
| **SX10** | **VI** | **-** | **N** | **-** | **-** | **-** | **-** | **-** | **-** | **-** | **-** | **-** | **-** | **-** | **-** | **-** |
| **F48E9** | **IX** | **-** | **-** | **-** | **-** | **-** | **-** | **-** | **-** | **-** | **-** | **-** | **-** | **-** | **-** | **-** |

“-” indicates the same sites as LaSota. The different sites was marked in red.
